# Lung ultrasound: A comparison of image interpretation accuracy between curvilinear and phased array transducers

**DOI:** 10.1002/ajum.12347

**Published:** 2023-05-27

**Authors:** Michael H. Walsh, Leo M. Smyth, Janeve R. Desy, Ernest A. Fischer, Alberto Goffi, Na Li, Matthew Lee, Joëlle St‐Pierre, Irene W. Y. Ma

**Affiliations:** ^1^ Department of Medicine, Cumming School of Medicine University of Calgary Calgary Alberta Canada; ^2^ Department of Community Health Sciences University of Calgary Calgary Alberta Canada; ^3^ Division of Hospital Medicine, Department of Medicine MedStar Georgetown University Hospital Washington District of Columbia USA; ^4^ Interdepartmental Division of Critical Care Medicine and Department of Medicine University of Toronto Toronto Ontario Canada; ^5^ St. Michael's Hospital and Li Ka Shing Knowledge Institute, Keenan Research Centre Unity Health Toronto Toronto Ontario Canada

**Keywords:** accuracy, image interpretation, lung ultrasound, point‐of‐care ultrasound, transducer

## Abstract

**Introduction:**

Both curvilinear and phased array transducers are commonly used to perform lung ultrasound (LUS). This study seeks to compare LUS interpretation accuracy of images obtained using a curvilinear transducer with those obtained using a phased array transducer.

**Methods:**

We invited 166 internists and trainees to interpret 16 LUS images/cineloops of eight patients in an online survey: eight curvilinear and eight phased array, performed on the same lung location. Images depicted normal lung, pneumothorax, pleural irregularities, consolidation/hepatisation, pleural effusions and B‐lines. Primary outcome for each participant is the difference in image interpretation accuracy scores between the two transducers.

**Results:**

A total of 112 (67%) participants completed the survey. The mean paired accuracy score difference between the curvilinear and phased array images was 3.0% (95% CI: 0.6 to 5.4%, P = 0.015). For novices, scores were higher on curvilinear images (mean difference: 5.4%, 95% CI: 0.9 to 9.9%, P = 0.020). For non‐novices, there were no differences between the two transducers (mean difference: 1.4%, 95% CI: −1.1 to 3.9%, P = 0.263). For pleural‐based findings, the mean of the paired differences between transducers was higher in the novice group (estimated mean difference‐in‐differences: 9.5%, 95% CI: 0.6 to 18.4%; P = 0.036). No difference in mean accuracies was noted between novices and non‐novices for non‐pleural‐based pathologies (estimated mean difference‐in‐differences: 0.6%, 95% CI to 5.4–6.6%; P = 0.837).

**Conclusions:**

Lung ultrasound images obtained using the curvilinear transducer are associated with higher interpretation accuracy than the phased array transducer. This is especially true for novices interpreting pleural‐based pathologies.

## Introduction

The role of bedside point‐of‐care ultrasound (POCUS) in assessing medical patients has been increasingly recognised.^1^, ^2^, ^3^ In particular, in assessing patients with dyspnoea, lung ultrasound (LUS) has been shown to be associated with higher diagnostic accuracy and a lower time‐to‐diagnosis when compared to traditional diagnostic approaches.^4^, ^5^, ^6^ For patients with heart failure, LUS use at hospital discharge can predict readmission at 6 months.^7^ Compared to chest radiograph, LUS has been shown to be more sensitive and specific for identifying pleural effusion^8^, ^9^, ^10^ and more sensitive for identifying pneumothorax.^11^, ^12^ Given this broad applicability, the ability for LUS to accurately detect findings such as B‐lines, pleural effusion, hepatisation and pneumothorax is of high interest, as quality LUS can assist with clinical management decisions. However, high diagnostic accuracy of LUS should not be assumed. There are several factors that impact the diagnostic accuracy of LUS, including but not limited to training and equipment factors. Prior studies have found that machine settings, such as depth, gain, focal position and use of tissue harmonic imaging, impact the ability to detect B‐lines.^13^, ^14^ Less is known about the impact of transducer type on LUS imaging.

For general LUS, a low‐frequency transducer is typically used. Existing training resources most commonly recommend the use of either a curvilinear transducer^15^, ^16^, ^17^ or a phased array transducer.^18^ In some references, no clear preference is stated between these two transducers.^19^, ^20^ Those that prefer the phased array transducer like its smaller footprint, which allows it to fit between the ribs.^21^ Conversely, those that prefer the curvilinear transducer like its larger footprint, which yields a wider field of view.^22^ To our knowledge, little empirical data to date support the recommendation of one transducer over the other. Our study seeks to compare, amongst internal medicine physicians and trainees, the interpretation accuracy of LUS images obtained using a curvilinear transducer with that obtained using a phased array transducer. We hypothesise that while equipoise may exist for using either transducer, diagnostic accuracy may depend on POCUS experience level of the image interpreter.

## Materials and methods

This is a cross‐sectional survey study, where participants with prior training in LUS were invited by email to complete an online survey (Qualtrics, Provo, UT). We invited 156 hospitalists, specialists or subspecialists in internal medicine, and trainees in internal medicine who were known to the investigator team. Additional 10 participants were then identified by initially recruited participants and invited using snowball sampling. Of these, six participated. Participants with no prior LUS training and those who did not consent were excluded. The survey was administered to participants between October 2020 and January 2021.

The survey included eight paired images/cineloops of select LUS findings from eight patients (*i.e*. total 16 images/cineloops). These paired images were obtained at the same location on a patient's chest, using both a curvilinear array and a phased array transducers (described in more detail below). Images were presented to the participants in an online survey in a random order to avoid paired images being presented sequentially. Participants were asked to indicate the pathology/pathologies that they could identify in each image.

Between January and March 2020, two trained investigators (MW or LS) obtained LUS images from a convenience sample of consenting patients admitted to the general medical ward in a tertiary hospital (Foothills Medical Centre) in Calgary, AB, Canada. Included patients were those with radiographical or known clinical diagnoses to support the following target LUS findings of interest: normal lung (n = 1), pneumothorax (n = 2), pleural irregularities (n = 1), consolidation/hepatisation (n = 1), pleural effusions (n = 2) and B‐lines (n = 1).

For each of the above LUS findings, with the patient in the semi‐recumbent position, we obtained two sequential 6 s cineloops (or M‐mode still images, where relevant), at the same location on the patient's thorax using two different low‐frequency transducers (5–2 MHz curvilinear and 5–1 MHz phased array; X‐Porte, FUJIFILM Sonosite, Inc., Bothell, WA, USA). General setting, rather than resolution or penetration setting, was used in all images. To enhance artefact capture, images were captured with tissue harmonics imaging and multibeam artefact features turned off.

**Figure 1:**
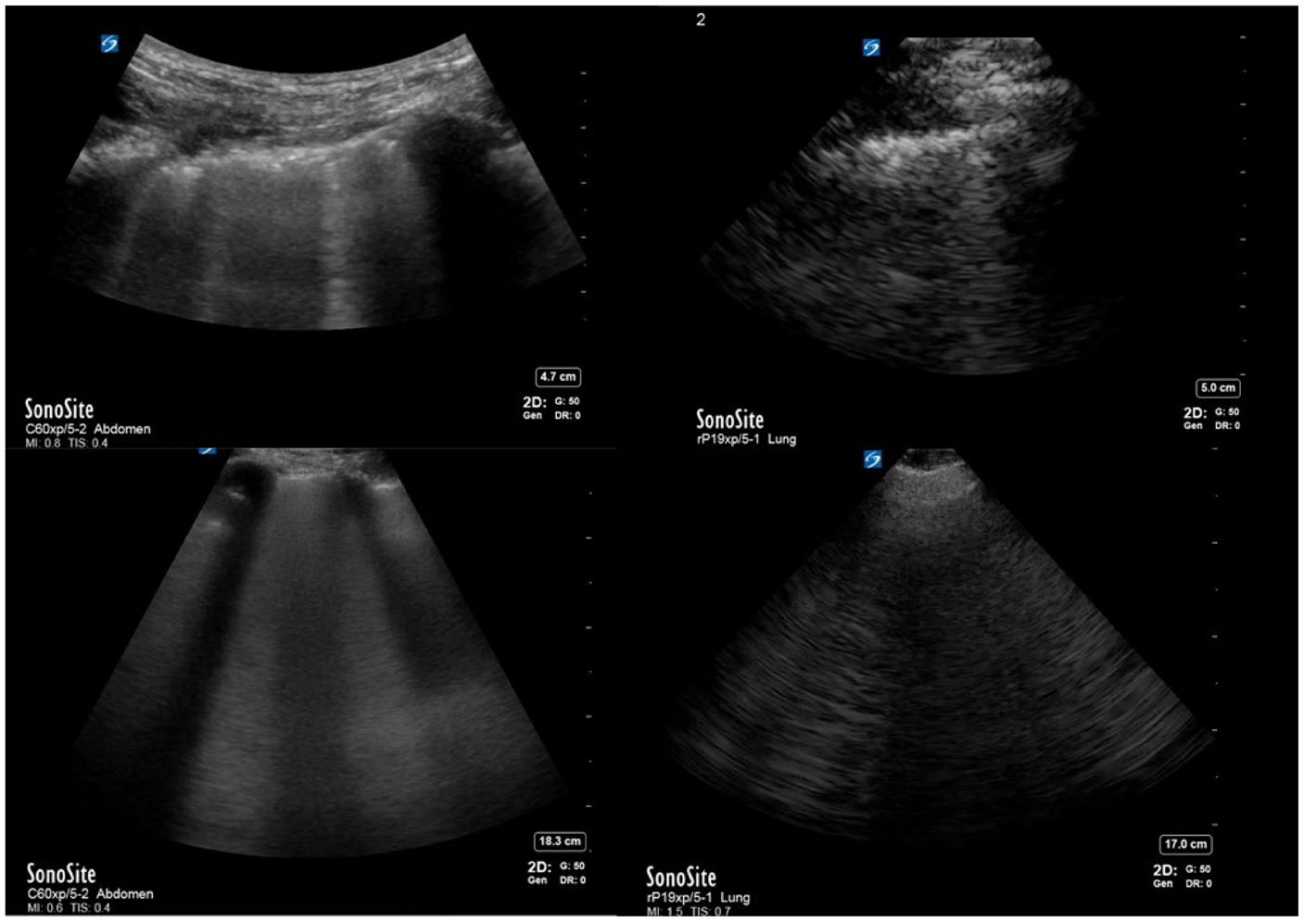
Representative still images taken from cineloops. Top left depicts the presence of irregular pleural line, comet tails (B‐ or Z‐lines) and subpleural consolidation on screen right, captured using a curvilinear transducer. Top right depicts the same pathology captured using a phased array transducer. Bottom left depicts the presence of B‐lines, captured using a curvilinear transducer. Bottom right depicts the same B‐lines captured using a phased array transducer.

The lung pre‐set was used for the phased array transducer. As no lung pre‐set was available for the curvilinear transducer, the abdominal pre‐set was used. Gain sliders (akin to time gain compensation) were set at mid‐point for both near and far field, with a gain setting of 50 for all images. Focal zone adjustment is not an option on our device. Therefore, manufacturer pre‐sets were used. Depending on the patient characteristics, depth was set between 4.7 and 7 cm for pleural‐based findings and 14.3 and 19 cm for non‐pleural‐based findings. The closest comparable depth was chosen between the curvilinear and phased‐array transducers (Figure 1). Both investigators who performed the LUS (MW and LS) were completing a 1‐year internal medicine POCUS fellowship and were at least 6 months into their training at the time of study.

A draft online survey with the above LUS findings was developed by two investigators (MW and IM). The appropriateness of survey items to include, as well as the clarity of the LUS images, was reviewed by an independent POCUS and medical education expert (JD), certified by the American Registry for Diagnostic Medical Sonography and has a Master's degree in Education in the Health Professions. After determining the appropriateness of survey items, she independently identified LUS findings to validate the intended LUS pathologies. This expert was blinded to the clinical diagnoses of the patients. Subsequently, four study authors (MW, LS, JD and IM) re‐reviewed the survey to ensure that survey clarity and flow were optimised. No coauthors responsible for survey construction or image acquisition completed the survey as a study participant.

### Study outcomes

Image interpretation in the identification of LUS findings for each image was rated out of a total score of two. A score of two was given if all findings were correctly identified, one if partial findings were correctly identified and zero if responses were incorrect. Overall accuracy score for each participant was presented as the number of points awarded divided by a maximum total possible of 32 points and presented as a per cent. For interpretation accuracy score of images obtained by the curvilinear transducer and phased array transducer, points were divided by a maximum total possible of 16 points, presented as a per cent. Our primary outcome for each participant is the difference between accuracy scores of the two transducers: curvilinear and phased array.

Secondary outcomes included accuracy score differences between pleural‐ and non‐pleural‐based images and between novice POCUS users compared to non‐novices. Pleural‐based images were those whose findings typically involved close observation of the pleural line (*e.g*. lack of lung sliding in pneumothorax, irregular pleura, with or without subpleural/peripheral consolidation). Non‐pleural‐based images were those whose findings involved evaluating both the pleura and the areas in the far field (*e.g*. pleural effusion, normal LUS findings, B‐lines and larger consolidation/hepatisation).

Non‐novice users were those who reported having completed a POCUS fellowship, at least 1 month of POCUS training, or those with a POCUS‐specific certification. All others with less intensive training were considered novice users.

### Statistical analysis

To detect a mean of paired differences of 5% between the accuracies for curvilinear and phased array images, assuming a standard deviation of the differences of 20%, to achieve a power of 80% and level of significance of 5%, a sample size of 128 participants is needed using a two‐sided one‐sample *t*‐test.

Standard descriptive statistics, including frequency (percentage) for categorical variables and mean (standard deviation; SD) or median (interquartile range; IQR) for continuous variables, are reported. Boxplots were graphed to illustrate the distributions of paired differences of accuracy scores. For the primary analysis, the mean of paired differences between the accuracies for curvilinear and phased array images for all participants was tested against zero using a two‐sided one‐sample *t*‐test. The mean and 95% confidence interval (CI) and the test P‐value are reported. A P‐value <0.05 was considered as statistically significant. Secondary analyses were performed using a two‐sample *t*‐test to compare the means between novice and non‐novice groups and using a paired *t*‐test to compare the means between pleural‐ and non‐pleural‐based images. Multivariate linear regression was used to compare the means of the paired accuracy differences for curvilinear and phased array transducer images between novice and non‐novice groups (the difference‐in‐differences analysis). All analyses were performed using SAS version 9.4 (SAS Institute Inc., Cary, NC, USA) and R version 4.1.1 (R Foundation for Statistical Computing, Vienna, Austria).

This study was approved by the Conjoint Health Research Ethics Board at the University of Calgary (REB 19–1181).

## Results

Of the 166 participants invited to complete the survey, 134 (81%) responded. Of these, 112 participants completed the survey (response rate 67%). Baseline characteristics of the participants are presented in Table 1. Of the 112 participants, 108 (96%) responded to the question on prior POCUS training. Of these, 50 participants were considered novices and 58 participants were considered non‐novices.

**Table 1:** Baseline characteristics of the 112 participants who completed the survey.

Baseline characteristic	N (%)
Sex	
Male	72 (64)
Female	40 (36)
Age	
<35 years	43 (38)
35–44 years	58 (52)
45–54 years	9 (8)
55–64 years	2 (2)
Country	
Canada	65 (58)
USA	43 (38)
Other	4 (4)
Training level	
Resident	30 (27)
Postgraduate year 1	0
Postgraduate year 2	9/30 (30)
Postgraduate year 3	5/30 (17)
Postgraduate year 4	8/30 (27)
Postgraduate year 5 or higher	8/30 (27)
Faculty	82 (73)
<5 years in practice	28/82 (34)
5–10 years in practice	39/82 (48)
11–20 years in practice	10/82 (12)
>20 years in practice	4/82 (5)
Prior point‐of‐care ultrasound training	
Novice	50/108 (46)
Non‐novice	58/108 (54)

For the 112 participants, the mean accuracy scores were 75.6% (95% CI: 72.7 to 78.6%) for curvilinear transducer generated images and 72.4% (95% CI: 69.7 to 75.1%) for phased‐array images (Table 2). The mean of paired differences between the accuracies for curvilinear and phased array images was 3.0% (95% CI: 0.6 to 5.4%; P = 0.015), which was significantly different from zero.

**Table 2:** Mean accuracy scores, presented as a per cent and 95% confidence interval in novices and non‐novices.

	Overall (n = 108)	Novices (n = 50)	Non‐novices (n = 58)
Curvilinear transducer images	75.6% (72.7 to 78.6%)	70.8% (65.8 to 75.7%)	79.8% (76.7 to 83.0%)
Phased‐array images	72.4% (69.7 to 75.1%)	65.4% (61.7 to 69.0%)	78.4% (75.1 to 81.8%)
Mean paired differences (curvilinear vs. phased array)	3.0% (0.6 to 5.4%, P = 0.015)	5.4% (0.9 to 9.9%, P = 0.020)	1.4% (−1.1 to 3.9%, P = 0.263)

### Secondary analyses

**Figure 2:**
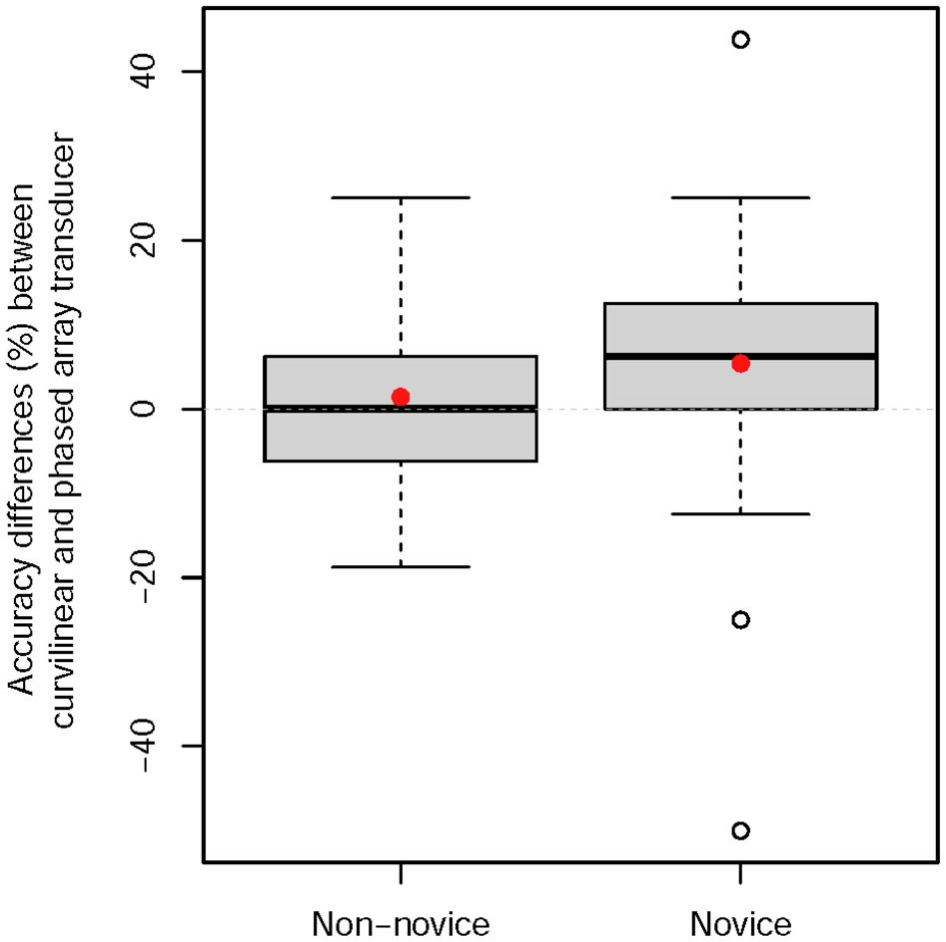
Box plots of interpretation accuracy score differences between curvilinear and phased array transducers in non‐novices and novices, where mean differences are indicated by a red dot.

Overall mean accuracy scores for novice participants were significantly lower than non‐novices (68.1%, 95% CI: 64.3 to 71.8% vs. 79.2%, 95% CI: 76.2 to 82.1%; P < 0.001, Figure 2). Mean accuracy scores for novices were higher on curvilinear transducer images than phased array images (mean difference: 5.4%, 95% CI: 0.9 to 9.9%, P = 0.020, Figure 2, Table 2). For non‐novices, there were no statistically significant differences in scores (mean difference: 1.4%, 95% CI: −1.1 to 3.9%, P = 0.263, Figure 2, Table 2).

Overall mean accuracy was lower for pleural‐based images than non‐pleural‐based images for the whole group (61.2%, 95% CI: 57.4 to 65.0%, for pleural‐based vs. 81.7%, 95% CI: 79.0 to 84.4% for non‐pleural‐based; mean difference: −20.5%, 95% CI: −24.3 to −16.7%, P < 0.001).

For pleural‐based images, using linear regression, adjusted for gender, the mean of the paired accuracy differences between curvilinear and phased array transducer images was significantly higher in the novice group than the non‐novice group (estimated mean difference‐in‐differences: 9.5%, 95% CI: 0.6 to 18.4%; P = 0.036). For non‐pleural‐based images, however, no significant difference in mean accuracies were noted between novices and non‐novices (estimated mean difference‐in‐differences: 0.6%, 95% CI: to −5.4–6.6%; P = 0.837).

## Discussion

In this study of 112 respondents out of 166 practicing internists, hospitalists and internal medicine trainees with reported prior training in LUS, interpreting images obtained using the curvilinear transducer demonstrated higher accuracy compared to the phased array transducer. The accuracy advantage from using a curvilinear transducer was only seen in novices, defined as those with less than 1 month of POCUS training or without POCUS certifications/fellowship training. Pleural‐based image interpretation appeared more challenging for the entire cohort than non‐pleural‐based images. Novices, in particular, had higher accuracy when interpreting pleural‐based images obtained using the curvilinear transducer than those obtained using the phased array transducer. These results suggest that for optimising image interpretation accuracy, images from curvilinear transducers may be superior, particularly for novices when interpreting pleural‐based images.

Curvilinear transducers have a larger footprint than phased array transducers. This larger footprint allows for better visualisation of near‐field structures and anatomic landmarks,^23^ which may explain our study findings, particularly with respect to pleural‐based image interpretation in novices. As previously stated, some resources do recommend the use of the curvilinear transducer for general LUS.^15^, ^16^, ^17^ However, a number of commonly used existing resources recommend either the phased‐array transducer^18^, ^24^ or indicates no specific preferences.^19^, ^20^ Few existing studies evaluate the differences between the two transducers, and in those that do, a signal is present suggesting that the curvilinear transducer may be superior. In one study on pneumothorax evaluation, image quality was rated to be higher for the curvilinear than the phased array transducer, but a difference in diagnostic accuracy was not noted between the two transducers.^25^ In another study of LUS of dogs, the ability to detect B‐lines did not differ between the two transducers, but image quality score was significantly higher for the curvilinear transducer than the phased array transducer.^23^ Our study extends these results by including more pathologies and a larger number of image interpreters, with a broader range of LUS experience.

Our study has some limitations. First, we limited our comparison to curvilinear and phased array transducers. We did not evaluate the use of linear transducers. For the diagnosis of pneumothorax, the linear transducer is generally agreed upon as the preferred transducer.^15^, ^19^, ^20^, ^26^ We also did not evaluate the use of microconvex transducers, recommended for the assessment of pleural effusion and pneumothorax,^19^ as this transducer was not available at our institution. Second, our study isolated the task of image interpretation from image acquisition and the clinical context. Potentially, the benefits seen in image interpretation accuracy using a curvilinear transducer may not be realised if clinicians were able to integrate relevant clinical information in real time. Third, despite our best attempt to choose the closest comparable depths between the two transducers, the depth set was not identical between the two transducers. Therefore, image accuracy differences seen may be a function of depth rather than the transducer type. Devices that allow identical depths to be set may help clarify this issue. Further, for the phased array transducer, we used a lung pre‐set, which was not available for the curvilinear transducer on our device. Potentially, the inclusion of a configured lung pre‐set by the manufacturer for the curvilinear transducer may further increase the accuracy differences seen in our study. Study conclusions may differ if different pre‐sets were used. In addition, study conclusions may also differ in a different commercially available device than ours, as devices may differ in terms of their available pre‐sets and any customisable image optimisation features such as focal zones, compound imaging and tissue harmonics. Until there is a universal consensus on LUS pre‐sets, POCUS users are encouraged to optimise their devices by working with the ultrasound manufacturers. Fourth, with no prior data to guide us, our sample size calculation was performed using an SD of 20%. With this, we estimated that we needed 128 participants and stopped recruitment at 134. However, upon data analysis, we realised that only 112 participants actually completed the surveys. Therefore, our study is underpowered. However, given that our observed SD is actually lower than anticipated, repeat sample calculation suggests that we only require 73 participants to detect a mean of paired differences of 5%. In addition, in our sample size calculation, our group felt that a score difference of 5% (or missing 1 question out of 16) is of concern, given that all LUS findings included in our study were those that we felt were clinically relevant. Whether or not this 5% difference is of clinical consequence, however, is unknown. Fifth, because of the multi‐centred nature of our study, we were not able to capture how participants were taught LUS. Potentially, greater accuracy seen with one transducer over another is simply a reflection of existing training practices, which currently are disparate and not standardised. Our study survey did ask participants at the start of the study, which transducer they preferred for performing LUS. However, this question was introduced when data collection was already underway and therefore did not systematically capture responses from all participants. However, 49 of the 50 novices did respond to this question. Of the 49 novices, 21 (43%) preferred using the curvilinear transducer, 22 (45%) preferred the phased array and 6 (12%) stated no preferences. These results suggest (but cannot confirm) that likely novice participants were taught to use both transducers. Lastly, the response rate of our survey was 67%. Although there is no universally agreed upon acceptable response rate, acceptability generally ranges between 50% and 75%.^27^, ^28^ Therefore, our response rate is not out of keeping with existing survey studies, but a higher response rate would have been preferred. In addition, our convenience/non probabilistic sampling strategy may introduce selection bias, which may affect generalisability of our results.

These limitations notwithstanding, our study findings do highlight a few messages that may be of importance to LUS educators. Specifically, LUS images obtained using the curvilinear transducer are associated with a higher interpretation accuracy than those obtained using the phased array transducer. This image interpretation advantage is primarily observed amongst novices interpreting pleural‐based pathologies. Therefore, we recommend the use of curvilinear transducers in general LUS (where possible), especially by novices, to optimise diagnostic accuracy. If this is not possible, when reviewing findings with learners, educators should pay close attention to the interpretation of pleural‐based pathologies, as these may be more likely to be misidentified by novices. Alternatively, POCUS users can also optimise their devices by working with the ultrasound manufacturers to customise their pre‐sets.

## Author contributions

**Michael H Walsh:** Conceptualization (equal); data curation (lead); investigation (equal); methodology (equal); project administration (equal); resources (equal); validation (equal); writing – original draft (equal); writing – review and editing (equal). **Leo M Smyth:** Conceptualization (equal); investigation (equal); writing – review and editing (equal). **Janeve R Desy:** Conceptualization (equal); investigation (equal); writing – review and editing (equal). **Ernest A Fischer:** Data curation (equal); investigation (equal); writing – review and editing (equal). **Alberto Goffi:** Data curation (equal); investigation (equal); writing – review and editing (equal). **Na Li:** Formal analysis (lead); methodology (equal); software (lead); validation (lead); writing – review and editing (equal). **Matthew Lee:** Data curation (equal); investigation (equal); writing – review and editing (equal). **Joëlle St‐Pierre:** Data curation (equal); investigation (equal); writing – review and editing (equal). **Irene W Ma:** Conceptualization (lead); data curation (equal); formal analysis (equal); funding acquisition (lead); investigation (equal); methodology (equal); project administration (equal); resources (equal); software (supporting); supervision (lead); validation (equal); visualization (equal); writing – original draft (equal); writing – review and editing (equal).

## Funding

This study is supported by the John A. Buchanan Research Chair in General Internal Medicine, University of Calgary. The funder had no role in the design, data collection and analysis, decision to publish, or preparation of the manuscript.

## Conflict of interest

IM is supported by the John A. Buchanan Chair in General Internal Medicine, University of Calgary. AG has received speaker honoraria from Philips Healthcare. The remaining authors have no conflicts of interest to declare.
